# Measuring Animal Age with DNA Methylation: From Humans to Wild Animals

**DOI:** 10.3389/fgene.2017.00106

**Published:** 2017-08-17

**Authors:** Ricardo De Paoli-Iseppi, Bruce E. Deagle, Clive R. McMahon, Mark A. Hindell, Joanne L. Dickinson, Simon N. Jarman

**Affiliations:** ^1^Institute for Marine and Antarctic Studies, University of Tasmania Hobart, TAS, Australia; ^2^Australian Antarctic Division Hobart, TAS, Australia; ^3^Sydney Institute of Marine Science Sydney, NSW, Australia; ^4^Cancer, Genetics and Immunology Group, Menzies Institute for Medical Research Hobart, TAS, Australia; ^5^Trace and Environmental DNA Laboratory, Department of Environment and Agriculture, Curtin University Perth, WA, Australia; ^6^CSIRO Indian Ocean Marine Research Centre, University of Western Australia Perth, WA, Australia

**Keywords:** epigenetics, ageing, methylation, wild animals, conservation, ecology

## Abstract

DNA methylation (DNAm) is a key mechanism for regulating gene expression in animals and levels are known to change with age. Recent studies have used DNAm changes as a biomarker to estimate chronological age in humans and these techniques are now also being applied to domestic and wild animals. Animal age is widely used to track ongoing changes in ecosystems, however chronological age information is often unavailable for wild animals. An ability to estimate age would lead to improved monitoring of (i) population trends and status and (ii) demographic properties such as age structure and reproductive performance. Recent studies have revealed new examples of DNAm age association in several new species increasing the potential for developing DNAm age biomarkers for a broad range of wild animals. Emerging technologies for measuring DNAm will also enhance our ability to study age-related DNAm changes and to develop new molecular age biomarkers.

## Introduction

Biological ageing involves complex interactions of accumulating organ, cellular, and DNA damage leading to functional decline and increased risk of death (Fontana et al., 2010). Biological functions including the age of reproductive maturity (Charpentier et al., 2008; Jones et al., 2014), reproductive frequency (Froy et al., 2013), and mortality (Pérez-Barbería et al., 2014) can be better understood in the context of age. Estimates of chronological age are therefore useful for understanding these key ecological characteristics of wild animals. However, chronological age is difficult to estimate in individuals of most animal species (Nussey et al., 2013). Many species lack measurable external features that change with age. Therefore, new methods that allow estimation of chronological age will enhance our understanding of ageing and population biology in wild animals.

DNA methylation (DNAm) at cytosine guanine dinucleotides (CpGs) is the best studied epigenetic modification and can repress gene expression when associated with gene promoters (Jones et al., 2015). CpG methylation in mammals is regulated by DNA methyltransferases (DNMTs). DNMTs are required for the initial establishment of methylation patterns in early development (Law and Jacobsen, 2010); and for maintaining established patterns of DNAm over the lifespan of the animal (Jones and Liang, 2009). There are two types of age-associated DNAm in vertebrates, “epigenetic drift” and “clock-type” DNAm (Jones et al., 2015). DNMT1 is primarily responsible for maintaining CpG methylation and its decline in activity with age is thought to contribute to a decrease in global methylation or “drift” in ageing cells (Jones et al., 2015). However, gene-specific DNAm change with age may be regulated by other de-novo DNMTs, such as DNMT3b (Lopatina et al., 2002). “Clock-type” age-associated DNAm is a change in methylation proportion (either an increase or decrease) at specific CpG sites. Changes at clock-type CpGs may be related to functional changes in gene expression with age (Horvath, 2013; Steegenga et al., 2014).

In this mini-review, we summarise current knowledge of observed age-related changes of CpG DNAm in mammals, reptiles, birds and fish. Recent technological advances have enabled the relatively quick analysis of large numbers of CpG loci (Parle-Mcdermott and Harrison, 2011). This has greatly increased the number of studies that have observed age-related DNAm in humans and model organisms (Jarman et al., 2015). We also describe how this new information can be used to develop molecular age biomarkers (MABs) for non-model animals. We explore environmental and behavioural studies of DNAm that are relevant to age estimation. We also discuss emerging technology and their potential for application in wild animals.

## DNA methylation ageing signals in humans and mice

**Figure 1:**
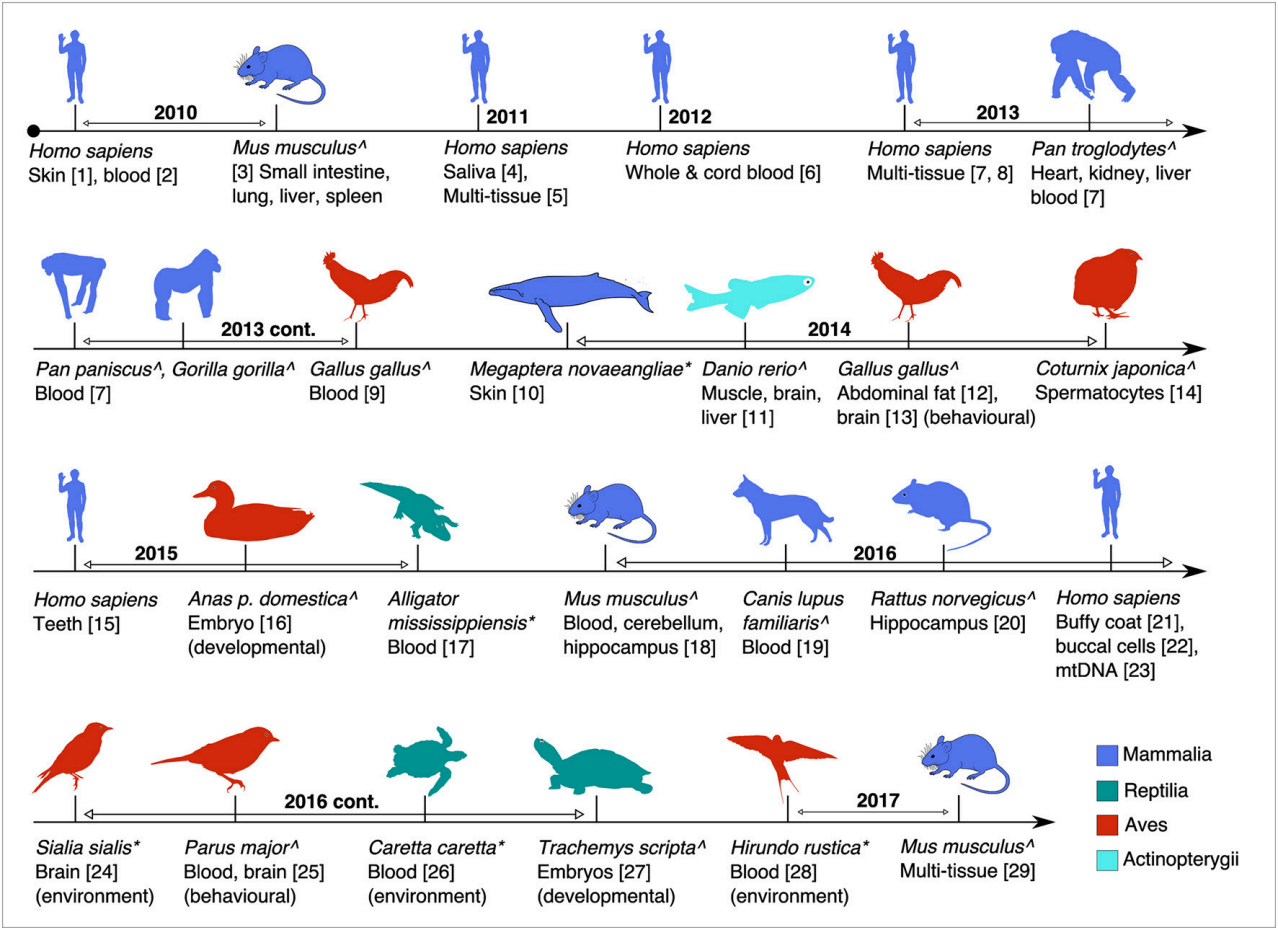
Timeline of the major studies and tissues analysed for global or targeted DNA methylation in this review. Studies are age-associated except where indicated. Superscripts: ^∧^(captive raised or model studies) and ^*^(wild animal studies). (1, Grönniger et al., 2010; 2, Teschendorff et al., 2010; 3, Maegawa et al., 2010; 4, Bocklandt et al., 2011; 5, Koch and Wagner, 2011; 6, Garagnani et al., 2012; 7, Horvath, 2013; 8, Hannum et al., 2013; 9, Gryzinska et al., 2013; 10, Polanowski et al., 2014; 11, Shimoda et al., 2014; 12, Sun et al., 2014; 13, Nätt et al., 2014; 14, Andraszek et al., 2014; 15, Bekaert et al., 2015; 16, Yan et al., 2015; 17, Nilsen et al., 2016; 18, Spiers et al., 2016; 19, Gryzinska et al., 2016; 20, Penner et al., 2016; 21, Christiansen et al., 2016; 22, Eipel et al., 2016; 23, Mawlood et al., 2016; 24, Bentz et al., 2016; 25, Verhulst et al., 2016; 26, Caracappa et al., 2016; 27, Matsumoto et al., 2016; 28, Romano et al., 2017; 29, Stubbs et al., 2017).

Age estimation models based on CpG DNAm combine information from CpG sites that have the highest correlation of DNAm levels with age. These are calibrated using tissue samples from known-age individuals. Early ageing models for humans used single tissues and small numbers of CpG sites (Bocklandt et al., 2011). Recent more precise models predict age from multiple tissues (Horvath, 2013). DNAm changes with age can contribute to altered gene expression levels during normal ageing (Zykovich et al., 2014) and disease (Nilsson et al., 2014). Therefore, the effect of CpG DNAm on transcriptional regulation of gene expression has been the focus of intense research (Goyns, 2002; Figure 1). The first studies to identify human age-related DNAm changes studied monozygotic twins, where epigenetic drift with age was observed when comparing older and younger twins (Fraga et al., 2005). This observation was supported by further studies of monozygotic twins and healthy controls that led to the first epigenetic age models (Boks et al., 2009). A detailed summary of age-related DNAm studies is shown in Table S1.

Age-related DNAm changes have been identified in several human and mouse tissues. Analysis of human skin samples for DNAm changes identified a set of CpG sites that were affected by chronological age. Thirty sun-exposed and sun-protected skin biopsy samples were analysed using the Infinium HumanMethylation27 (27 K) BeadChip (Illumina). The study identified 104 CpG sites that had a DNAm relationship with age, but not by sun exposure (Grönniger et al., 2010). Another example of age-related CpG DNAm was found in the saliva samples of 34 identical twins (21–55 years old). This study identified 88 CpG sites where DNAm levels were significantly correlated with chronological age (27 K BeadChip) (Bocklandt et al., 2011). The genes identified were involved in age-related cardiovascular and neurological diseases (Park et al., 2007; Bocklandt et al., 2011). In mice, linear age-related methylation was identified in multiple CpG sites from 12 different genes measured in intestine, lung, liver, and spleen. This indicated that similar age-related changes in DNAm levels can be found in humans and other mammals (Maegawa et al., 2010).

Most clock-type DNAm changes are tissue specific, however several studies have investigated age-associated changes in multiple tissues. Non cell-type dependent DNAm changes were identified in a study that combined several published CpG DNAm data sets to predict age using multiple tissues (Koch and Wagner, 2011). Four CpG sites in *TRIM58, KCNQ1DN, NPTX2*, and *GRIA2* were identified from a set of 431 hypermethylated CpGs. Multiple linear regression of the DNAm levels for each CpG site against the known donor age resulted in a model with a mean absolute difference (MAD) of ±10.3 years (Koch and Wagner, 2011). Another multi-tissue model based on 353 CpGs was developed from 82 publicly available data sets of 8,000 healthy tissue and cell types and had a median absolute difference of ±3.6 years (Horvath, 2013). This study predicted age using a greater range of tissue types and show that cancer can lead to an increased age as measured by DNAm. The model was more accurate in heterogeneous tissues such as blood and saliva compared to breast tissue, dermal fibroblasts, and skeletal muscle. The author suggested hormonal or cancer effects as possible causes of this variation. Horvath (2013) proposed that this model measures the cumulative work of an epigenetic maintenance system that likely functions over the entire mammalian lifespan. The DNA methylome and human ageing rates were also compared using a far greater number of CpG sites, the HumanMethylation450 (450K) BeadChip (Hannum et al., 2013). Here, a MAB using 71 CpGs predicted chronological age with a MAD of ±3.9 years. In mice, a multi-tissue age predictor has been developed using 329 CpG sites, giving a median absolute error of ±3.3 weeks (Stubbs et al., 2017). Together, these three models show that methods based on large numbers of markers can improve precision of age estimates in model organisms or humans.

Lifestyle factors can influence DNAm levels in humans and mice and could impact chronological age estimation if not corrected for. A positive difference between estimated DNAm age and known age suggests that an individual is biologically older than their chronological age. Described epigenetic clocks have been used to show decreased age acceleration dependent on diet (Quach et al., 2017) and increased age acceleration associated with smoking (Zaghlool et al., 2015; Gao et al., 2016) and body mass index (Horvath et al., 2014). Recent research has also highlighted the differences between human and murine DNAm clocks (Wagner, 2017) and the effects of calorie restriction on mouse biological age (Petkovich et al., 2017).

The use of DNAm biomarkers for specialised forensic applications generally involves using fewer CpG sites in simpler assays. A model based on three CpG sites in human blood yielded a MAD from known-age samples of ±5.4 years, which was an improvement over other non-epigenetic molecular ageing techniques (Weidner et al., 2014). In two studies, a small number of CpG sites in one gene region (*ELOVL2*) allowed simplification of technical analyses while maintaining prediction accuracy (MAD ± 3.9 years) (Zbieć-Piekarska et al., 2015a,b). Single multiplex reactions such as methylation-sensitive single-nucleotide primer extension can be used to make age biomarkers that are cheaper to run than pyrosequencing or microarray assays. For example, one study using this method with eight CpG sites led to age predictions with a MAD of ±6.07 years (Vidal-Bralo et al., 2016). While this approach had lower precision than previous studies it is still a feasible tool for estimating age using adult blood. One study implemented the models published by both Horvath (2013) and Hannum et al. (2013). Here, buffy coat was isolated from twins (30–82 years) and age was predicted (Christiansen et al., 2016). This resulted in MADs of ±5.6 years for the 353 CpG Horvath model and ±5.4 years for the 71 CpG Hannum model demonstrating the benefit of using a higher number of CpG sites.

Methylation changes in mitochondrial DNA (mtDNA) associated with age have been identified in humans. An epigenetic model based on two CpGs with a MAD of ±9.3 years was developed from the blood of 82 individuals (18–91 years). Age was correlated with mtDNAm at two sites, M1215 and M1313, in the 12s *MT-RNR1* gene (Mawlood et al., 2016). mtDNA overall has a low level of CpG methylation (2–6%), so detection of age-related CpG levels required high assay precision. This is the only study of age-related mtDNA methylation and it is still uncertain whether mtDNAm-based models will match the accuracy of models using genomic biomarkers.

## Quantifying DNA methylation, environmental effects and age in model and wild animals

### Mammals

DNAm age biomarkers have only been developed for a small number of wild mammal species. In long-lived species, obtaining known-age calibration sample sets that cover the entire lifespan is a significant obstacle. DNAm age estimation is most advanced in species closely related to humans. The age of chimpanzees (*Pan troglodytes*), bonobos (*Pan paniscus*), and gorillas (*Gorilla gorilla*) were estimated using the 353 CpG clock MAB created for humans (Pai et al., 2011; Hernando-Herraez et al., 2013; Horvath, 2013). Results from blood samples showed that in both chimpanzees and bonobos the model had an accuracy similar to that found in humans; however, accuracy was reduced in gorillas (Horvath, 2013).

Humpback whales (*Megaptera novaeangliae*) have successfully been used as a test case for applying knowledge of human age-related clock type DNAm change to estimate age in a long-lived wild mammal. Forty-five known-age samples were used to calibrate a DNAm age model. Seven of 37 CpG loci screened by pyrosequencing showed significant age-related DNAm. The three sites with the strongest relationship with age were used to predict whale age from skin with a MAD of 3.75 years. This model also predicted the correct order of ages in samples with known kinship in more than 93% of cases (Polanowski et al., 2014).

Global DNAm levels in dogs change with age as a result of epigenetic drift. Significant differences in relative global DNAm levels have been found amongst pups (43.5%), adolescents (53.6%), adults (61.5%), and old dogs (81.2%) (Gryzinska et al., 2016). Clock-type DNAm age biomarkers based on multiple CpG sites have been developed for dogs. These models were calibrated using blood from multiple known-age animals and could predict age with a minimum MAD of 23.1 months (Ito et al., 2017).

### Birds

**Figure 2:**
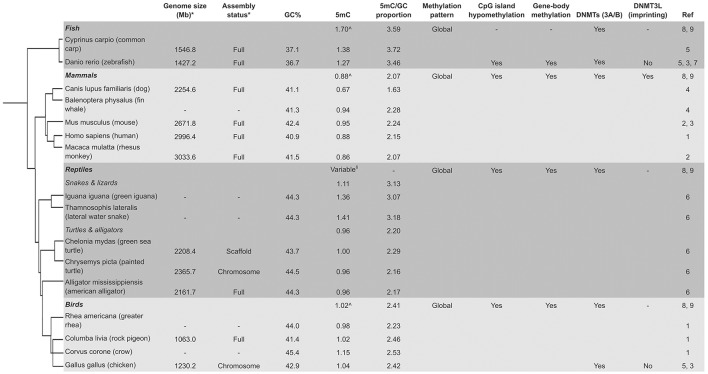
Variable global methylation in vertebrates. ^*^Current genome on NCBI (if available). ^∧^Average 5 mC for classes (Jabbari et al., 1997). (1, Ehrlich et al., 1982; 2, Gama-Sosa et al., 1983; 3, Yokomine et al., 2006; 4, Jabbari et al., 1997; 5, Vanyushin et al., 1970, 1973; 6, Varriale and Bernardi, 2006; 7, Shimoda et al., 2014; 8, Ponger and Li, 2005; 9, Okamura et al., 2010; Tree, Letunic and Bork, 2006).

DNAm patterns in birds are relatively unexplored compared to mammals (Head, 2014). Most avian DNAm research focuses on chickens (*Gallus gallus*) and quails (*Coturnix japonica*). Observation in *G. gallus* of unmethylated CpG islands in gene promoters (Li et al., 2011) and altered CD4 gene transcription due to increased DNAm of the promoter, indicates a similar regulatory function to that in mammals (Luo et al., 2011; Figure 2). An age-related decrease in percentage DNAm of six CpG sites in the *PPAR*γ promoter in 2, 3, and 7-week-old *G. gallus* has also been reported (Sun et al., 2014). Global DNAm levels have been shown by immunoenzymatic assay to decrease with age in *G. gallus* (Gryzinska et al., 2013). Higher global DNAm levels of 55-week-old hens (*G. gallus*) compared to 20-week-old individuals have recently been shown in breast tissue using whole-genome bisulphite sequencing. Of 2,714 identified differentially methylated regions, 378 were mapped to gene promoters including *ABCA1, COL6A1*, and *GSTT1L*. CpG sites in these genes were hypermethylated with age and could be used for future age biomarker studies (Zhang et al., 2017). DNAm analysis of *C. japonica* DNA by gel imagery showed that 15-week-old quails had increased DNAm of the *RN28S* gene compared to 52-week-old individuals (Andraszek et al., 2014).

DNAm studies of behavioural traits in several bird species could yield potential targets for age biomarker development if the identified genes are linked to behavioural change during an animal's life. The DNAm level in the dopamine receptor D4 (*DRD4*) gene in great tits (*Parus major*) was shown to be associated with variations in exploratory behaviour (Verhulst et al., 2016). Methylation tiling arrays have linked the promoters of the zinc finger RNA binding protein (*ZFR*) gene and male hypermethylated region (*MHM*) with sex dependent gene expression in chicken brain samples (Nätt et al., 2014). Several of the differentially expressed genes were known to affect behaviours including exploration and fearfulness.

Age-associated global DNAm could be affected by environmentally altered DNMT expression in birds. Increases in mRNA for DNMT1, DNMT3A, and methyl binding protein MBD5 were found after a 1°C increase in incubation temperature in several tissues of embryonic Peking ducks (*Anas platyrhynchos domestica*). These changes in expression levels of methylation-interacting enzymes could lead to overall changes in DNAm of bird genomes with age (Yan et al., 2015). A single CpG in the *ER*α promoter in wild eastern bluebirds (*Sialia sialis*) was positively correlated with yolk testosterone concentration and nestling growth rate. While DNAm at this CpG appears to depend on maternal and environmental conditions, it may help to better estimate age in pre-fledgling chicks (Bentz et al., 2016).

The epigenetic effect of adverse environmental conditions has recently been analysed in a wild barn swallow (*Hirundo rustica*) population. After particulate matter exposure, DNAm levels in two *Clock* gene loci were significantly increased in chicks (7 to 5 days old) and mothers. This study is a good example of a targeted DNAm approach and is the first study to show that DNAm levels can change in response to anthropogenic pollutants in wild birds (Romano et al., 2017). Exposure to DNAm altering compounds could be used to produce DNAm age biomarkers if exposure is consistent over time and among individuals in a population.

### Reptiles

Reptile DNAm has not been well studied in general and there is little data on age-related DNAm. Reptilian CpG island positions relative to promoters are similar to those found in mammals and birds, indicating that DNAm has a similar regulatory function (Varriale and Bernardi, 2006; Head, 2014). Adult American alligators (*Alligator mississippiensis*) have consistently lower global DNAm than sub-adults and captive juveniles (Parrott et al., 2014). The decrease in global DNAm through epigenetic drift is consistent with that found in all other studies to date (Nilsen et al., 2016). While there are no age-related clock-type reptilian studies, some have measured changes in DNAm due to environmental influences. Reduced global DNAm due to phenotypic differences was found in the loggerhead sea turtle (*Caretta caretta*) (Caracappa et al., 2016). In the red-eared slider turtle (*Trachemys scripta*) CpG DNAm levels of the *aromatase* gene were associated with shifts in egg incubation temperature (Matsumoto et al., 2013, 2016).

### Fish

Fish DNAm is better studied compared to birds and reptiles, with the majority of research on the zebrafish model (*Danio rerio*). Zebrafish embryos show high levels of global CpG methylation (80%), which is similar to mouse (74%), and also have depletion of methylation around transcriptional start sites similar to mammals (Feng et al., 2010). However, there are important differences in epigenetic reprogramming during early embryogenesis that are reviewed elsewhere (Potok et al., 2013; Head, 2014; Figure 2). A gradual and clear loss of CpG DNAm using methylation-sensitive enzymes and cloning was shown in 3, 18, and 30-month-old zebrafish (Shimoda et al., 2014). As with other non-model animal groups, there is little DNAm age data for wild fish.

## Future directions

Changes to DNAm patterns have been established as biomarkers of chronological and biological ageing in humans and have great potential for age estimation in wild animals. Global DNAm hypomethylation correlating with age has been found in a range of wild animals, suggesting that epigenetic drift occurs in most vertebrates. Numerous clock-type age-related CpG sites have also been identified, several of which appear to be well conserved in mammals (Table S1). Both epigenetic drift and clock-type DNAm changes could be used for age estimation in vertebrates.

New technologies for measuring changes in DNAm relating to age will improve our ability to generate age biomarkers. Nanopore technology has recently improved so that it is possible to identify cytosine and adenosine methylation variants in *E. coli* (Rand et al., 2017). An advantage of nanopore technology is that relatively small amounts of non-treated DNA are required to produce long sequence reads compared to bisulphite treated DNA. This may be advantageous for animal studies where DNA yield from the target tissue is low, such as feather quill ends (Simpson et al., 2017). Digital restriction enzyme analysis of methylation (DREAM) allows the precise measurement of CpG sites in a global context (Jelinek et al., 2012; Maegawa et al., 2014). Preparation of samples for both methods is relatively simple and does not require a reference sequence to identify age related signals (Jelinek and Madzo, 2016).

Measuring wild animal age with DNAm has diverse applications in ecological and environmental research. Populations of known-age wild animals will be particularly important for this type of research. Age estimates generated from a robust DNAm model could be used to understand survival, reproductive potential, and biological ageing (Jarman et al., 2015; Jazwinski and Kim, 2017). Development of age biomarkers in new species will benefit immensely from information on age-related DNAm change gathered from humans and model organisms. This field is poised to change the way that the age of wild animals is determined.

## Author contributions

Conception: RD, JD, SJ; Design: RD, SJ, BD, MH, CM; Drafting original: RD, SJ, BD; Critical revision: JD, MH, CM; Final approval and accountability, all authors.

### Conflict of interest statement

The authors declare that the research was conducted in the absence of any commercial or financial relationships that could be construed as a potential conflict of interest.
